# Quantitative assessment of neural elements in a rat model using nerve growth factor after remnant-preserving anterior cruciate ligament reconstruction: a histological and immunofluorescence pilot study

**DOI:** 10.1186/s13018-020-01792-6

**Published:** 2020-07-23

**Authors:** Sung Hyun Lee, Hyung Gyu Cho, Jin Soo Song, Keun Churl Chun, Churl Hong Chun

**Affiliations:** 1 Department of Orthopedic Surgery, Wonkwang University Hospital, 895, Muwang-Ro, Iksan, 54538 South Korea; 2 Department of Biological Sciences, College of Natural Sciences, Wonkwang University, Iksan, South Korea; 3 Department of Orthopedic Surgery, Hankook Hospital, Mokpo, South Korea

**Keywords:** Anterior cruciate ligament, Remnant preservation, Immunofluorescence, Nerve growth factor

## Abstract

**Background:**

Immunofluorescence analyses of anterior cruciate ligament (ACL) allografts following remnant-preserving ACL reconstruction using Achilles tendon allografts have provided evidence for the presence of neural elements. In this study, we aimed to examine the expression of neural elements and quantify the presence of neural cells in ACL remnants and Achilles allografts using nerve growth factor (NGF) therapy after remnant-preserving ACL reconstruction.

**Methods:**

Experiments were conducted on 5 pairs of rats (approximately 8 weeks old and weighing 320 g at the time of surgery). Longitudinally, split Achilles tendons from the paired rats were freshly frozen and later defrosted with warm saline and allografted onto the right ACL of the other rat that was partially detached at the femoral attachment site. A sham operation was conducted on the left knee to be used as a control. NGF was injected into both knee joints every week for 6 weeks after surgery. The presence of neural cells in the ACL of the sham-operated knee, allografted Achilles tendon, and ACL remnant was examined 6 weeks post-surgery using H and E and immunofluorescent staining.

**Results:**

H and E staining did not reveal neural cells in any of the three groups. However, immunofluorescence analysis showed the presence of nestin-positive neural elements in the normal ACL tissues as well as ACL remnants. Additionally, neural elements were examined in 7 of the 8 (87.5%) allograft tissues. Quantitative analysis showed no difference in the number or area of nuclei among the three groups. However, the number and area of neural cells in the Achilles allografts were significantly lower than those in the other two groups (*p* = 0.000 and *p* = 0.001, respectively).

**Conclusion:**

Our observations indicate that ACL remnants promote the new ingrowth and persistence of neural cells. We suggest that the ingrowth of neural elements can support the persistence and new ingrowth of mechanoreceptors, thereby enhancing the functional stability of knee joints. Moreover, the expression of neural cells in the Achilles allografts was lower than that in normal ACL tissues and ACL remnants in the quantitative evaluation, thereby confirming the essential role of ACL remnants in knee joint functionalization.

## Background

The functional instability of a knee joint is primarily caused by the lack of coordinated muscle stabilization, thereby inciting pertinent interest in unravelling methods to protect mechanoreceptors in the knee joint [1, 2]. It has been reported that the remnant-preserving ACL reconstruction technique preserves mechanoreceptors and renders positive results [1, 3–7]. The current study aimed to determine whether the remnant preservation technique preserves the existing mechanoreceptors or leads to re-innervation.

Evidence has demonstrated the growth of mechanoreceptors in reconstructed ACLs. For instance, Barrack et al. reported an increase in mechanoreceptors in ACL grafts in dogs 6 months after surgery compared to their normal patellar tendon [8]. Denti et al. reported that mechanoreceptors were present in the bone-patellar tendon autografts in the knees of sheep 3 months after surgery and in human knees with failed autografts at 9 and 10 years after surgery [9]. These findings suggest that ACL remnants are a possible source of re-innervation of the tendon graft. However, previous histological studies have failed to validate the presence of mechanoreceptors in ACL allografts [10]. In addition, few studies have quantitatively evaluated neural elements in ACL remnants and ACL allografts.

Nerve growth factor (NGF) is widely used clinically for its significant roles in supporting neuronal survival, peripheral nerve growth, nutritional adaptation, nerve regeneration, and fracture repair [11]. However, it has not been delineated whether these receptors affect re-innervation or proprioception. However, the persistence of mechanoreceptors after remnant-preserving ACL reconstruction is important. Thus, by stimulating the expression of neural cells using NGF, we tried to validate the superiority of the remnant preservation technique.

The aim of the present study was to investigate the effects of administering NGF following the remnant preservation technique by assessing the presence of nerve cells via immunofluorescence in the allograft and remnant ACL. In addition, neural elements in ACL remnants and ACL allografts were quantitatively evaluated using ImageJ particle analysis. We hypothesized that remnant tissues following ACL reconstruction as well as Achilles allografts would contain neural cells, which were identified using immunofluorescence.

## Methods

### Animals

Experiments were conducted on 10 adult male rats (*Rattus norvegicus albinus*, Samtako®, Korea) that were approximately 8 weeks old and weighed 320 g at the time of surgery. All the rats were numbered using ear tags. The rats were housed in accordance with the National Institutes of Health guidelines; they were kept in a vivarium, maintained at 20–25 °C and 60% humidity with 12 h alternating light–dark cycles (7 am–7 pm), and were provided food and water ad libitum.

### Surgical procedure

#### The remnant-preserving ACL allograft

**Fig. 1:**
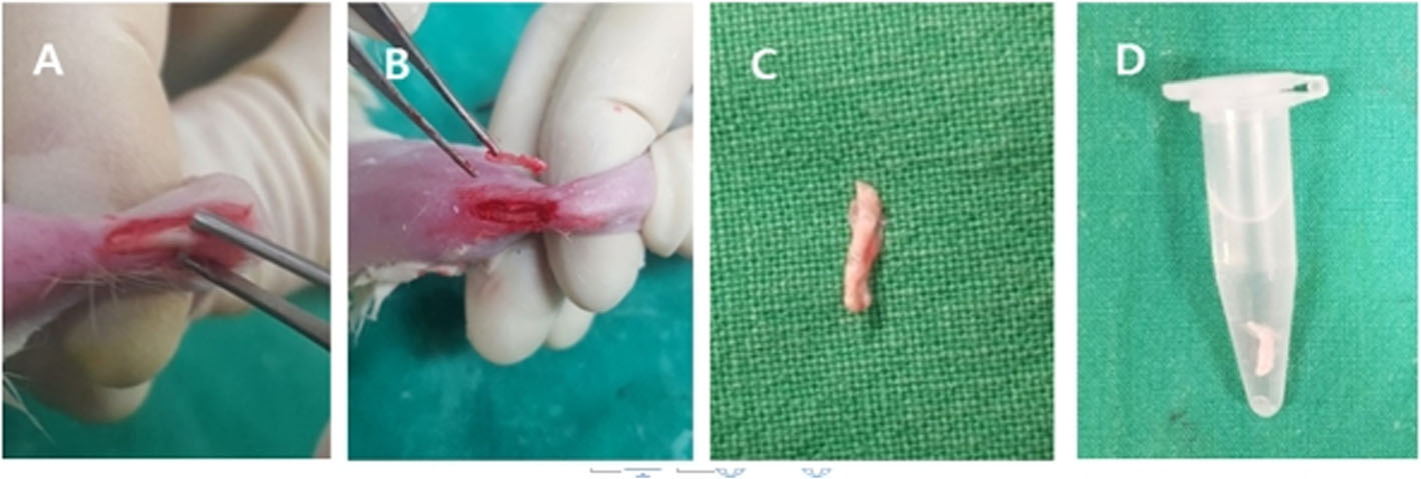
Achilles tendon was grafted for ACL reconstruction. **a** Achilles tendon exposure. **b**, **c** Split Achilles tendon. **d** Frozen Achilles tendon thawing in warm saline (47 °C for 5 min)

Arthrotomy was performed after intraperitoneal (IP) administration of general anaesthesia [a mixture of Zoletil (50 mg/kg, Virbac Laboratories, France) and Rompun (10 mg/kg, Bayer, Korea)]. In pairs, the Achilles tendon was obtained from a donor rat and used as an allograft for ACL reconstruction in the right knee of the recipient rat. Briefly, an approximately 1.5 cm longitudinal skin incision was made on the Achilles tendon insertion site in the left ankle. After longitudinally splitting 1.5 cm of the Achilles tendons of each rat, the tissue was fresh frozen at − 80 °C for 5 min in a deep freezer. Then, the Achilles tendon was defrosted with 47 °C warm saline for 5 min (Fig. 1) and used as an allograft.

**Fig. 2:**
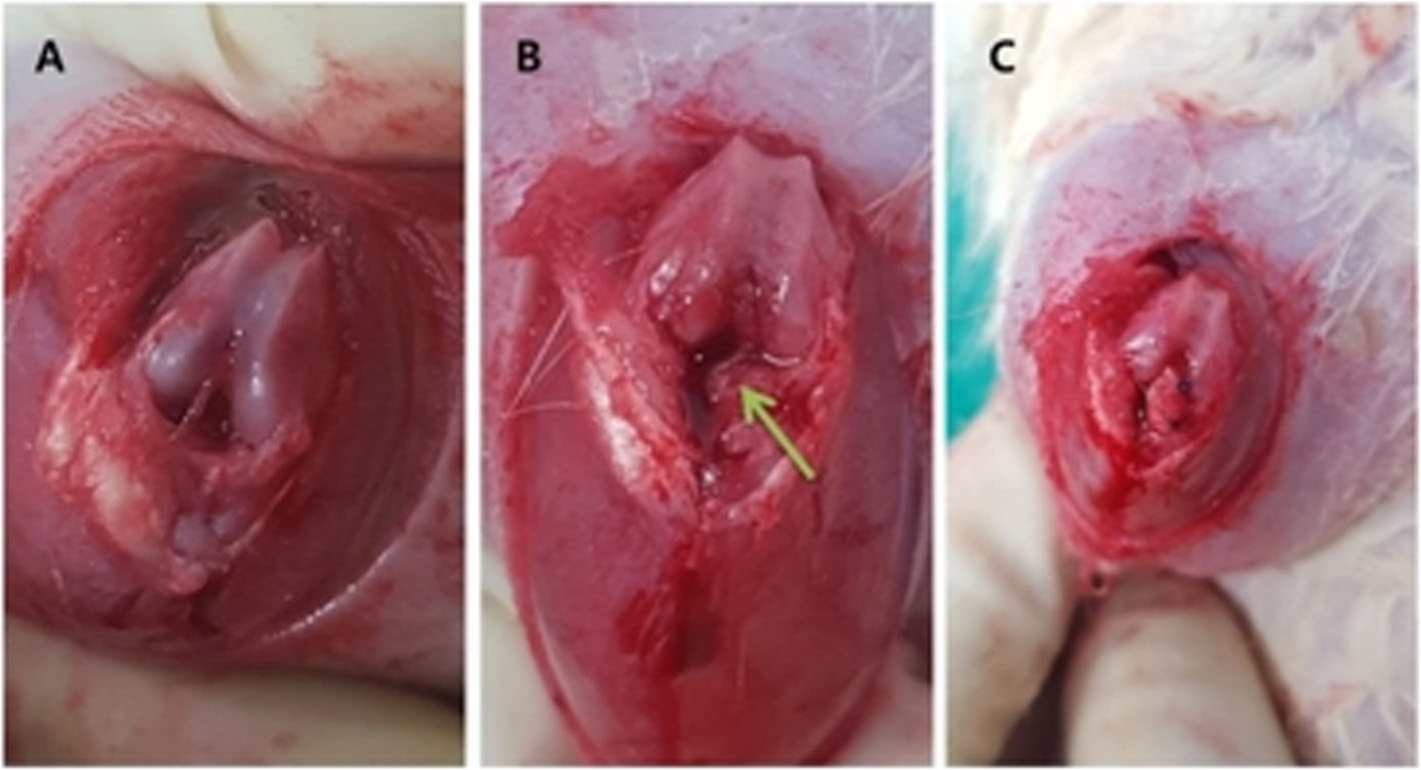
Achilles tendon was allografted to each rat’s left ACL. **a** ACL exposed. **b** ACL partially detached (green arrow). **c** Achilles tendon grafted to the ACL

For the ACL reconstruction, an approximately 1.5-cm incision was made on the right knee. Then, the joint was exposed by transposing the patella laterally after a parapatellar incision was made with the knee in flexion. The ACL was detached at the femoral insertion, but the tibial insertion was maintained. Subsequently, the Achilles tendon was allografted onto the right ACL, which was partially detached at the femoral attachment site. Suture fixation was used on the femoral and tibial ACL anatomical attached sites (Fig. 2). Irrigation was performed on the joints, and the capsule and skin were closed with interrupted sutures.

### The sham operation

A sham operation was conducted on the left knee as a control. The knee joints were exposed with the same incision. Additionally, irrigation was performed on the joints, and the capsule and skin were closed with interrupted sutures.

### NGF injection and sample collection

A total of 10 μl of recombinant rat NGF (50 μg dissolved in 500 μl ddH_2_O, Invitrogen #50385MNAC50, CA, USA) was injected into both knee joints every week for 6 weeks after the operation using a 0.3-cc insulin syringe with a microfine needle. The presence of neural cells in the control group (sham operation), allografted Achilles tendon, and ACL remnants was examined 6 weeks post-surgery using haematoxylin and eosin (H and E) staining and immunofluorescent staining with anti-nestin antibody. The presence of neural cells was then compared among the Achilles allografts, ACL remnants, and normal ACL (sham operation) tissues.

### H and E staining

Tissue samples were collected from the femoral insertion site of normal ACL tissues, the detached site of ACL remnants and the femoral insertion site of Achilles allografts. After dehydration with alcohol and washing with a tissue processor (Leica TP 1020, Leica, Germany), tissue pieces were fixed with masked formalin solution (mask form 2A, DANA Korea) for 24 h and embedded in paraffin. The tissues were sectioned sequentially into 4 μm thick slices and stained with H and E. Mechanoreceptors were classified into four types according to a prior study (type I, a spherical or ovoid Ruffini corpuscle; type II, a columnar concentric circular Pacini corpuscle; type III, a spindle-shaped Golgi corpuscle; and type IV, a non-myelinated free nerve ending) [12]. Sections with H and E staining were assessed for the presence of mechanoreceptors following the stated classification.

### Immunofluorescent staining

Sliced tissues on coverslips were washed three times with phosphate-buffered saline (PBS), fixed with 4% paraformaldehyde in PBS for 10 min, and permeabilized with 0.1% Triton X-100 in PBS for 5 min at room temperature. After washing three times with PBS, sections were blocked with 1% bovine serum albumin (BSA) for 1 h at room temperature. Sections were incubated with anti-nestin antibody (N5413, Sigma-Aldrich, St. Louis, MO) (diluted in blocking solution (1% BSA in PBS)) for 1 h at room temperature in a shading box. Subsequently, the tissue sections were washed three times with PBS and incubated with secondary antibody, Alexa Fluor 555-conjugated rabbit anti-goat antibody (Invitrogen, Grand Island, NY) for 1 h at room temperature. Alexa Fluor 488-conjugated phalloidin (Invitrogen, Grand Island, NY) was used for F-actin staining. Nuclei were stained with 4,6-diamidino-2-phenylindole (DAPI, Santa Cruz Biotechnologies, Santa Cruz, CA). Images were captured using a confocal microscope (Olympus, FV-1 mm).

### Quantification with particle analysis

After immunofluorescent staining, quantitative analysis was performed on each image using ImageJ (National Institutes of Health, Bethesda, MD). The number and area of nuclei that were stained with DAPI and the neural cells that were stained with anti-nestin antibody were calculated. Several attached cells in each image were optically separated using a watershed separation tool provided by the ImageJ software. Following this, the area and number of cells were quantified using ImageJ particle analysis.

### Statistical analysis

Statistical analysis was conducted using SPSS for Windows, version 12.0. The Kruskal-Wallis test was used to analyse the immunofluorescence data (with the 95% confidence level). *P* values < 0.05 were considered significant. Where indicated, Mann-Whitney post hoc analysis was performed after the Kruskal-Wallis test, with a significance level set at *p* < 0.16. The power of group comparison was analysed using G*Power 3.1.9.2, where this study achieved a power of 0.68 for detecting differences with *α* = 0.05.

## Results

Two of 10 rats expired during the experiment (at 2 weeks and 4 weeks post-surgery). The experiment was completed with the remaining 8 rats.

**Fig. 3:**
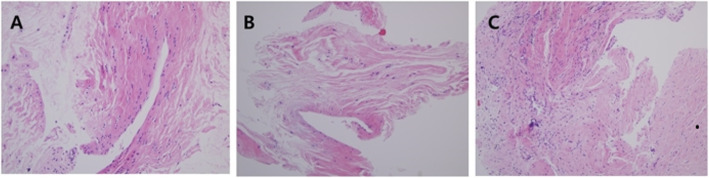
In all cases, no mechanoreceptors were present on H and E staining. **a** Normal ACL (control specimen). **b** Remnant ACL specimens. **c** Achilles allograft specimens (H and E stain, × 20)

H and E examination confirmed ligamentous ACL tissues and synovium in all the sections (Fig. 3). However, neural cells or mechanoreceptors could not be detected in normal ACL tissues, ACL remnants, or Achilles allograft tissues through H and E staining (Fig. 3).

**Fig. 4:**
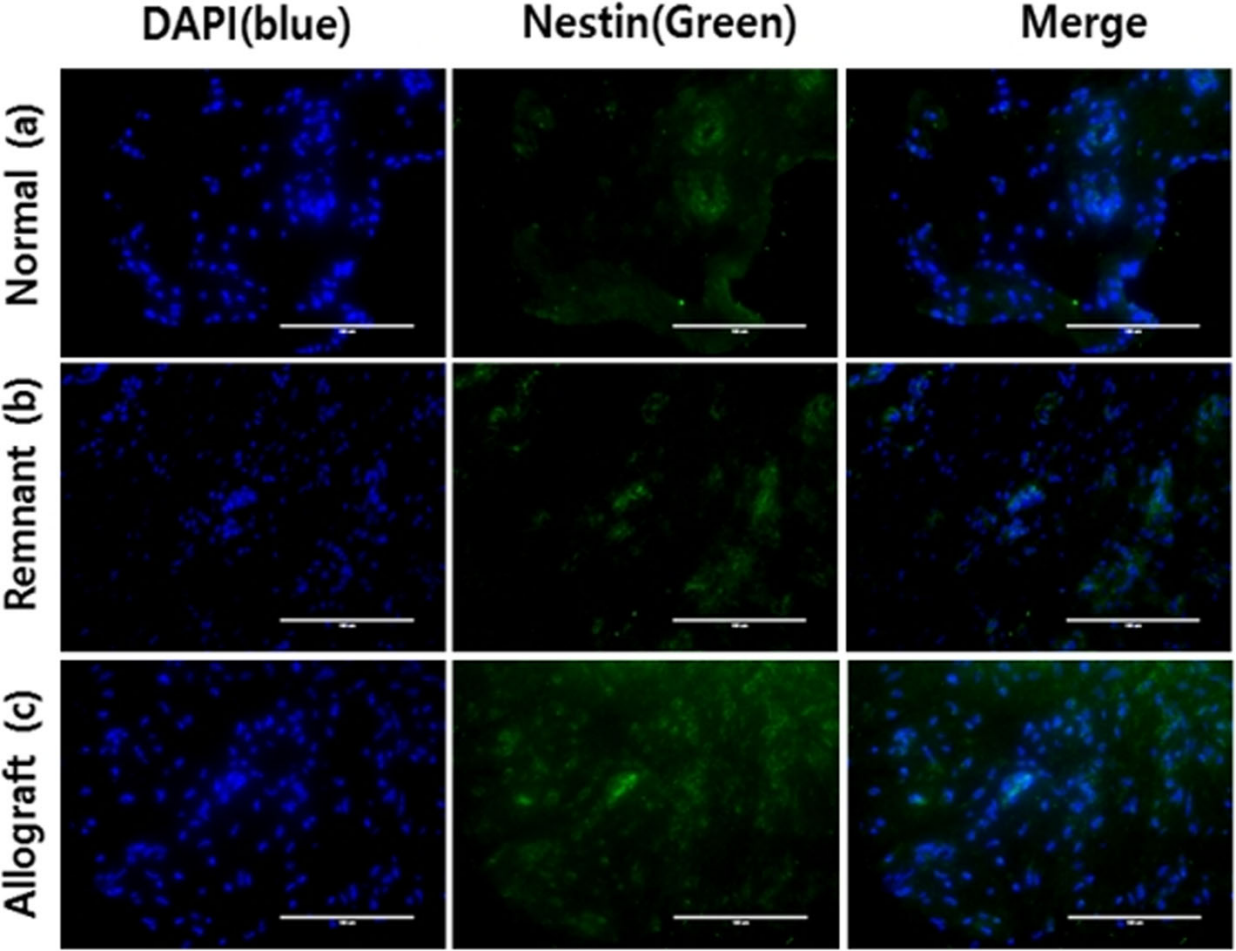
Immunohistochemical findings of nerve cells. Nuclei were stained with 4,6-diamidino-2-phenylindole (DAPI, Santa Cruz Biotechnologies, Santa Cruz, CA). Nerve cells were stained with anti-nestin antibody in **a** normal ACL tissues, **b** remnant ACL specimens, and **c** Achilles allograft specimens

In the immunofluorescence study, nestin was expressed in all normal ACL tissues that were injected with NGF. In addition, nestin was expressed in all ACL remnants and in 7 of 8 (87.5%) allograft tissues that were processed with NGF (Table 1). Nestin-positive cells suggested the possible presence of neural cells in the ACL remnant tissue and Achilles allografts (Fig. 4). We did not observe any significant difference in neural cell expression among the normal ACL tissues, ACL remnants, and Achilles allografts within the immunofluorescence study (*p* = 0.368).

**Table 1:** Immunofluorescence study on the presence of nestin-positive neural cells

Number	Presence of neural cells (nestin staining)		
	Normal ACL (sham operation)	Remnant ACL	Achilles allograft
#1	+	+	+
#2	+	+	+
#3	+	+	+
#4	+	+	+
#5	+	+	−
#6	+	+	+
#7	+	+	+
#8	+	+	+

+expression of nestin

−no expression of nestin

**Fig. 5:**
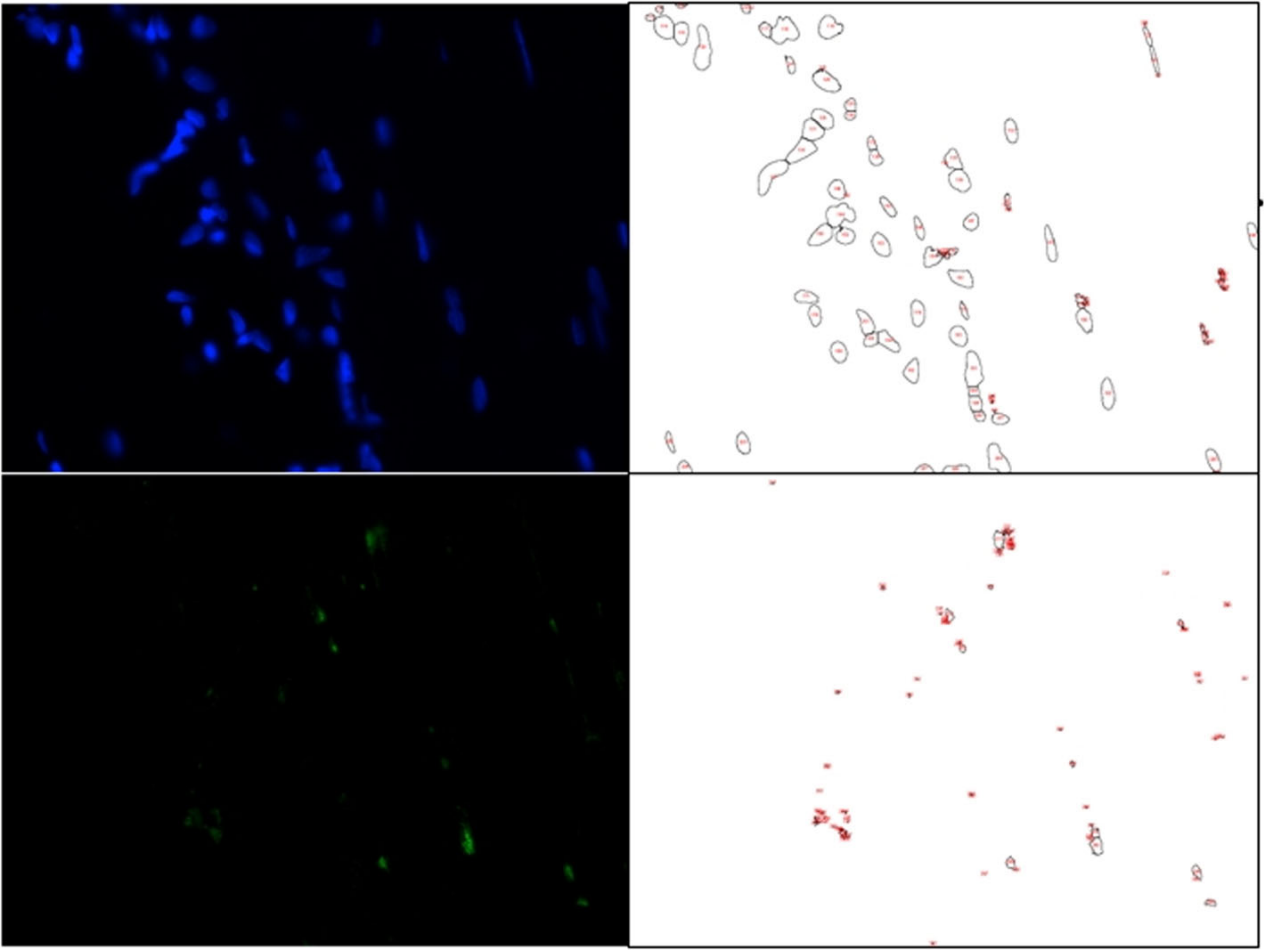
Several attached cells in each image were separated using the watershed separation tool based on the ImageJ software. Then, the area or number of cells was measured with ImageJ particle analysis

Quantitative analysis showed no difference in the number and area of nuclei among normal ACL tissues, ACL remnants, and Achilles allografts. However, the number and area of neural cells were significantly different among the groups. Post hoc analysis revealed that the number and area of neural cells in Achilles allografts were smaller than those in normal and remnant ACL tissues (*p* = 0.000 and *p* = 0.001, respectively) (Table 2) (Fig. 5).

**Table 2:** Number and area of nuclei and neural cells in normal, remnant, and allografted ACL tissues

		Normal ACL (*n* = 8)	Remnant ACL (*n* = 8)	Achilles allograft (*n* = 8)	*p*
Nuclei	Number	178.6 ± 84.3	114.4 ± 49.8	179.3 ± 78.7	0.227
	Area (pixels)	77565.9 ± 56207.1	41082.9 ± 24237.0	76713.6 ± 37395.3	0.138
Neural cell	Number	1977.3 ± 1521.8	1668.5 ± 1015.6	89.9 ± 68.8	0.000
	Area (pixels)	36218.9 ± 33157.0	29613.4 ± 21547.3	1846.1 ± 1179.4	0.001

Data are presented as the mean ± SD

## Discussion

This study used immunofluorescence analysis to shed light on a critical aspect of the localization of neural elements. We provide evidence that neural elements are found in not only remnant ACL tissues but also Achilles allografts. In the quantitative test, the neural cells of ACL remnants were not found to be different from those of normal ACL tissues. These results indirectly demonstrate that ACL reconstruction with remnant preservation can better preserve proprioception and be a resource for re-innervation in reconstructed allografts.

Proprioception is a key factor in maintaining the stability of the knee joint against functional deficiencies after ACL reconstruction. Many mechanoreceptors known to be distributed in ACL and remnant tissues contribute to the proprioceptive function of the knee [13, 14]. Some previous studies have reported the presence of mechanoreceptors in ACL remnants after ACL reconstruction with the remnant preservation technique [5, 15–17]. A previous study reported the presence of mechanoreceptors in ACL remnants 3 years after ACL rupture [17]. In another recent study, normal proprioceptive fibres and mechanoreceptors were reported in 50% of remnant ACL tissues using neurofilament protein staining [16]. They also showed a difference between mechanoreceptors in healthy and injured ACLs and highlighted their clinical significance [15]. Lee et al. reported the presence of mechanoreceptors in approximately one third of ACL remnants, which was less than expected [5]. In this study, neural cells were identified in ACL remnants, which was not different from normal ACL tissues upon quantitative evaluation. This outcome suggests that remnant-preserving ACL reconstruction can preserve proprioception and have a positive effect on knee joint functions.

Conversely, few studies have suggested the possibility that ACL remnants can serve as a source of re-innervation for reconstructed allografts. Ochi et al. stressed that restoring knee function is important in terms of anatomical ACL reconstruction, and that mechanical restraint and graft sensory re-innervation could potentially improve the overall outcome [18]. Denti et al. found re-innervation of autologous bone-patellar tendon-bone grafts in animals 3–6 months after surgery [9]. However, few studies have suggested the possibility that ACL remnants can serve as a source of re-innervation for reconstructed allografts. Kim et al. proposed that no new ingrown mechanoreceptors are present in Achilles allografts [10]. In addition, Chun et al. suggested that Achilles allograft ligaments do not show similar findings compared with biopsy samples from normal ACL tissues [19]. Recently, Chun et al. identified the presence of neural elements in ACL remnant tissues after remnant-preserving ACL reconstruction using immunofluorescence evaluation with NGF application. However, in their study, neural elements were not observed in Achilles allograft tissues [20]. Therefore, we investigated the expression of mechanoreceptors in remnant tissues and allografts following remnant-preserving ACL reconstruction using NGF therapy. In the present animal study, the presence of neural elements was confirmed in remnant tissues and allografts using immunofluorescence methods with NGF, a well-known growth factor for nerve cell proliferation.

The presence of neural cells in Achilles allografts supports the potential regeneration of neural elements during remnant-preserving ACL reconstruction. This was, however, not supported in previous studies [10, 19].

Many studies have evaluated proprioception, as well as the function of mechanoreceptors in reconstructed ACLs [5, 10, 13–17]. However, it is difficult to compare outcomes between these studies because of variations in the experimental methods and other external factors. Recently, immunofluorescence and immunohistochemistry using specific antigen-antibody reactions have been used to detect nerve fibres, producing more reliable and relevant results [5]. In this study, we visualized mechanoreceptor-positive cells using immunofluorescence assessments with monoclonal antibodies against nestin. To identify the presence of nestin-positive cells in the remnant tissues, the cells were treated in vivo with NGF, which is an important factor in the growth and maintenance of sensory and sympathetic neurons. Previous studies have reported that NGF application can be used to promote the healing of ACLs [21]. They proposed that NGF promoted re-innervation and angiogenesis in the healing of rat ACLs. Therefore, we evaluated the expression of mechanoreceptors in remnant tissues and allografts after remnant-preserving ACL reconstruction using in vivo administration of NGF in rats. The results of the current study indicate that NGF promotes re-innervation of Achilles allografts.

Few studies have attempted to quantify the proprioceptive potential of the injured ACL stump [9, 16, 17]. However, none of these studies have quantified their immunofluorescence findings. We analysed the number and area of nuclei and neural cells that were each stained with DAPI and anti-nestin antibody. The expression of neural cells in the Achilles allograft was less than that in normal ACL tissues and ACL remnants in the quantitative evaluation, although nerve growth was promoted by NGF.

There has been controversy regarding whether the superiority of remnant-preserving ACL reconstruction observed in immunofluorescence and immunohistochemical studies on proprioception can also be observed in terms of clinical function [20]. Some previous studies have reported that the remnant-preserving technique does not show more clearly favourable improvement in clinical outcomes than non-remnant-preserving techniques [22, 23]. In another recent study on a different ligament, there were also no significant differences in the management of chronic lateral ankle instability using a semitendinosus tendon autograft regardless of preservation of the remnant anterior talofibular ligament [24]. Therefore, to investigate the clinical significance of the expression of small amounts of neural elements in allografts, further research on the differences in proprioceptive function after remnant-preserving ACL reconstruction using allografts will be required.

As a limitation, the present study had a small sample size for determining satisfactory outcomes; however, since this was an experimental study, it was possible to perform experiments with a small sample size, and the limitation in detecting a difference was reduced by statistical analysis. We assessed only immunofluorescence analysis except for functional outcomes and sensory recoveries through the behavioural analysis of rats because the subjectivity of the experimenter could be involved. Additionally, because a functional evaluation was not performed, the sham operation consisting of only skin incision and closure was conducted on the left knee as a control; we considered that this simple procedure would not interfere with biomechanical loading and behaviour. As mentioned above, the lack of a biomechanical test was the major limitation of this study, but it might be thought to be observed in the form of partial integration or adhesion because the ACL was partially detached during surgery, and suture fixation was performed on the remnant tissue. Additionally, since the number of ACL samples collected from rats was too small and the slides were made with a thin thickness of 4 μm in the course of the experiment, it might be difficult to definitively confirm the results of H and E staining. Finally, after the injection of NGF, the presence of neural cells was confirmed by immunofluorescence using an anti-nestin antibody, but it was not possible to confirm whether these nerve cells absolutely matched the mechanoreceptors.

## Conclusion

The results from this study demonstrate that ACL remnants promote the new ingrowth and persistence of neural cells. We suggest that the ingrowth of neural elements can support the persistence and new ingrowth of mechanoreceptors and the functional stability of knee joints. However, the expression of neural cells in the Achilles allografts was lower than that in normal ACL and remnant ACL tissues in the quantitative evaluation.

## Data Availability

The datasets used and analysed during the current study are available from the corresponding author upon reasonable request.

## Abbreviations

ACL: Anterior cruciate ligament
NGF: Nerve growth factor
PBS: Phosphate-buffered saline
BSA: Bovine serum albumin
DAPI: 4,6-Diamidino-2-phenylindole
